# Changes in macular pigment optical density after membrane peeling

**DOI:** 10.1371/journal.pone.0197034

**Published:** 2018-05-14

**Authors:** Mario R. Romano, Gilda Cennamo, Piergiacomo Grassi, Federica Sparnelli, Davide Allegrini, Giovanni Cennamo

**Affiliations:** 1 Eye Clinic, Department of Bioscience, Humanitas University, Rozzano, Milan, Italy; 2 Eye Clinic, Department of Neuroscience, University Federico II, Naples, Italy; Massachusetts Eye & Ear Infirmary, Harvard Medical School, UNITED STATES

## Abstract

**Introduction:**

To highlight the differences in macular pigment optical density (MPOD) between eyes with vitreoretinal interface syndrome and healthy control eyes, to assess the changes in MPOD in eyes treated with macular peeling, to investigate the relationships between MPOD changes and measures of retinal sensitivity such as best corrected visual acuity (BCVA) and microperimetry.

**Methods:**

In this cross-sectional comparative study, 30 eyes affected by idiopathic epiretinal membrane (iERM, 15eyes) or full-thickness macular hole (FTMH, 15eyes) were compared with 60 eyes from 30 healthy age-matched patients. MPOD values (mean MPOD, maximum MPOD, MPOD area, and MPOD volume) were measured in a range of 4°–7° of eccentricity around the fovea, using the one-wavelength reflectometry method (Visucam 200, Carl-Zeiss Meditec). Patients affected by iERM and FTMH were treated with vitrectomy and epiretinal membrane-inner limiting membrane (ERM-ILM) peeling, with follow-up examinations performed preoperatively and 6 months postoperatively. The main outcome measures were the differences in MPOD between eyes with vitreoretinal interface syndrome and healthy eyes, changes in MPOD after ERM-ILM peeling, and relationships between MPOD and functional changes.

**Results:**

Mean MPOD differed significantly between control eyes and those with iERM (*P* = .0001) or FTMH (*P* = .0006). The max MPOD and MPOD area increased, but not significantly. After peeling, the only significant change in MPOD was in MPOD volume (*P* = .01). In the ERM group, postoperative mean MPOD correlated significantly with best-corrected visual acuity (*r* = .739, *P* = .002).

**Conclusions:**

MPOD was reduced in patients with iERM or FTMH compared with healthy eyes. We found a significant correlation between the mean postoperative MPOD and postoperative BCVA, hypothesizing that the postoperative increase in mean MPOD could be due to a change in distribution for unfolding and expansion of the fovea after the peeling. MOPD may be considered as a prognostic factor associated with a good visual prognosis in patients with iERM.

## Introduction

The typical yellow coloration of the macula is for the presence of macular pigment (MP) comprising the carotenoids lutein and zeaxanthin. MP is deposited preferentially in the fovea in the Henle fiber layer (comprising foveal cones’ axons) and in the parafovea in the inner plexiform layers of the retina,[1] where it absorbs energy-rich short-wavelength light. MP maximal concentration peaks is in the center of the macula (the foveola) and decreases progressively moving away from it.

The carotenoids play a key role in the antioxidant defense system, particularly against short wavelengths of visible light, because they absorb maximally at wavelengths of 460 nm and 440 nm (blue light). Blue wavelengths are more dangerous than longer wavelengths of visible light because they are more energetic and could be more efficient at producing reactive oxygen species from endogenous photosensitizers for example lipofuscin.[2,3] Therefore, high concentrations of lutein and zeaxanthin may protect against the development or progression of degenerative macular diseases.[4] Some studies have shown that healthy individuals have greater central MP values than those with AMD.[5–8]

Lutein and zeaxanthin are xanthophylls that are biochemically distinct from other carotenoids because of the hydroxyl groups at each end of these molecules, allowing them to be oriented in lipid membranes exposed to aqueous environments in a unique and possibly protective way. [2,9,10] Additionally, lutein and zeaxanthin are versatile antioxidants that neutralize reactive oxygen species in both the low-pO2 inner retina and the high-pO2 photoreceptor–retinal pigment epithelium (RPE) complex. This oxygen-rich outer retina is particularly vulnerable to oxidative damage because of the high concentrations of polyunsaturated fatty acids, which are susceptible to photooxidation and exposure to high-energy blue light.[2,3]

Macular pigment optical density (MPOD) is a measurement of the attenuation of blue light by MP and is linearly related to the amount (concentration × path length × area) of lutein and zeaxanthin in the macula if incorporated over the region where MP is deposited.^2^ Optical density levels, also called density units, typically met in the center of the human macula range between 0 and 1.[11,12]

There is considerable interest in measuring the amount and spatial distribution of macular carotenoids, with four main techniques: (i) flicker photometry, (ii) fundus autofluorescence images, (iii) Raman spectroscopy, and (iv) fundus reflectometry. In flicker photometry, the MPOD is taken as the log ratio of the perceived intensity of blue light in the center to that in the periphery.[13,14] It is the most commonly used technique, and does not need pupil dilation but does require a high degree of patient collaboration. Fundus autofluorescence measures MPOD levels according to the MP’s attenuation of fluorescent light arising from lipofuscin in the RPE, but cannot be used in a patient with cataracts.[15] Raman spectroscopy measures the vibration within molecules, which is proportional to the concentration of MP. However, it requires pupil dilation and is expensive.[5] Fundus reflectometry (used in this study) records images using blue (480–488 nm) and green (515–540 nm) wavelengths of light. The light traverses the MP twice. Because MP absorbs mainly blue light, subtracting the green and blue images after logarithmic transformation of the propagated light provides information about the distribution and quantification of MP.

The aims of this study were to examine the differences in MPOD between eyes with vitreoretinal interface syndromes (idiopathic epiretinal membrane (iERM) or full-thickness macular hole (FTMH)) and control eyes, to evaluate changes in MPOD in eyes with iERM and FTMH treated with epiretinal membrane-internal limiting membrane (ERM-ILM) peeling, and to assess whether MPOD and its changes might be considered as a possible prognostic factor to predict visual outcome in patients with iERM and FTMH who undergo surgery. To our knowledge, no studies in literature have reported the main changes in MPOD after surgical treatment in eyes with vitreoretinal interface syndromes, nor have any studies determined whether MPOD is correlated with functional outcomes. This could be of particular interest since, at present, only anatomic findings (such as the recovery of the ellipsoid zone after surgery) have been proposed in literature as possible prognostic factors correlated with the final functional outcome after ERM-ILM peeling.[16]

## Patients and methods

### Study design

This was a cross-sectional prospective comparative study of 30 eyes from 30 patients affected by iERM (15 eyes) or FTMH (15 eyes). These eyes were compared with 60 eyes from 30 healthy age-matched patients. We also investigated changes in MPOD after ERM-ILM peeling and examined the relationships between MPOD and anatomical or functional changes. Patients who underwent ERM-ILM peeling were followed up at 6 months after surgery.

The study and data collection methods received approval from the institutional review board of University Federico II of Naples. All patients were adults, written informed consent for the research and treatment was obtained from patients in adherence to the tenets of the Declaration of Helsinki.

### Protocol for assessing MP and analysis

The one-wavelength fundus reflectance method[17,18] was used (Visucam 200 Zeiss Meditec, Jena, Germany). The Visucam is a fundus camera that uses narrow-band wavelength reflectance to determine MP. Retinal areas containing MP absorb blue light more strongly than the rest of the retina. The reflectance spectrum at the fovea is attenuated at all wavelengths compared with that at the perifovea, particularly in the absorption range of the MP (400–550 nm of visible light).[19] In the blue reflectance image, the degree of darkening is a measure of the optical density of the MP.

Patients were asked to put their foreheads against the upper bar and chins on the chin rest to keep their heads still. Head alignment was maintained with chin-head straps and an internal fixation target with a central position was used for image alignment. The patients’ pupils were dilated with one drop of 1% tropicamide, and fundus color photographs at 45° were obtained 30 minutes after pupil dilation. As previously suggested by Delori et al., the illumination light [5.6 log photopic trolands (td) for 2–3 minutes] and the focusing light (6.8 log photopic td for 3–5 seconds) ensured that >99% of the cone pigments were bleached before the first excitation was used. For rods, respective illuminances of 4.7 and 6.1 log scotopic td for the same durations caused 59%–68% of the rhodopsin to be bleached before data collection commenced.[19] The retina was then illuminated with blue light, and only the blue channel of the capture sensor was used for the MPOD image—this suppresses unwanted autofluorescence signals in the green wavelength range. The MPOD was measured in a 30° field of the fundus photograph with the flash level set on automatic and flash intensity set at 12. Autofocus function was enabled. The MPOD signal was calculated in a range of 4°–7° of eccentricity around the fovea, spanning the region where the majority of xanthophylls are concentrated. This was separated from the background signal of the normal reflection of the retina beyond 7° of eccentricity, where it is assumed that no MP is present. The optical density and distribution of MP were calculated using a dedicated software algorithm, and the following fundus reflectometry measurements within each image were recorded: (a) mean MPOD and reproducibility; (b) maximum MPOD value; (c) MPOD area—the area within which the MP was detected and defined on the background; and (d) MPOD volume—the sum of all optical densities within the MPOD area. Mean MPOD is the ratio of volume to area, and referred to the mean optical density of the macular pigment xanthophylls in relation to the surface area. Maximum MPOD refers to the maximum optical density of the macular pigment xanthophylls (usually in the fovea). MPOD is measured in density units (du).[2,6]

The MPOD was calculated under the assumption that a_b_ is the absorption coefficient of the MP when excited by blue light and the Beer-Lambert law is applicable.

The calculation was performed using the following formulae with the reflection coefficient r for the retina and an optical density MPOD of the MP:

$$\begin{array}{l} {\text{I}}_{\text{P}}\;\left(\text{Intensity}\;\text{of}\;\text{reflected}\;\text{light}\;\text{from}\;\text{the}\;\text{perifoveal}\;\text{area}\right)={\text{r}\;\text{I}}_{\text{E}}\;\left(\text{Intensity}\;\text{of}\;\text{the}\;\text{blue}\;\text{excitation}\;\text{light}\right)\\ {\text{I}}_{\text{M}}\;\left(\text{intensity}\;\text{of}\;\text{the}\;\text{reflected}\;\text{light}\;\text{from}\;\text{the}\;\text{macular}\;\text{area}\right)={\text{r}\;\text{I}}_{\text{E}}\;\text{exp}\;\left(-2{\text{a}}_{\text{b}}\;\text{MPOD}\right) \end{array}$$

The division of the two equations produces the optical density MPOD:

$$\begin{array}{l} \text{MPOD}=\left[1/\left({\text{2a}}_{\text{b}}\;\text{lg}\;\text{e}\right)\right]\;\text{lg}\;\left({\text{I}}_{\text{P}}/{\text{I}}_{\text{M}}\right)\\ \text{MPOD}=\text{k}\;\text{lg}\;\left({\text{I}}_{\text{P}}/{\text{I}}_{\text{M}}\right) \end{array}$$

Whereby lg e is the logarithm of the basis 10.

The one-wavelength reflection method uses both the local and spectral selectivity of MP. Local selectivity means that MP is detectable only in a certain foveal region. Spectral selectivity means that MP absorbs blue light at wavelengths of <530 nm, maximally at 460 nm. The fundus is illuminated by blue light of one wavelength near the absorption maximum of MP. Under this illumination, simplistically, the fundus can be considered to be a uniform reflecting surface to which increased absorption in the fovea is added. The uniform reflection of the paramacular retina is violated by shading associated with retinal structures such as vessels (which show an increased absorption of the blue light) and exudates (which exhibit an increased reflection). One common way for shading correction is low-pass filtering, but only retinal pixels that do not contain any inhomogeneous structures such as vessels, exudates, drusen or MP itself should be used for shading correction.[20] The shading function of the central part of a fundus image can be approximated by a three-dimensional parabolic function. For the construction of such a paraboloid several nodes must be determined at fundus sites having no internal dishomogeneous structures. As previously suggested by Schweitzer et al, the shading function was calculated in an annular field centered on the fovea.[20] The inner diameter of this annulus was 1.2 times the diameter of the optic disk (PD) and the outer diameter was 2.4 PD. The shading function was assumed as the virtual background in the circular foveolar field, where the MPOD was determined. Assuming that the illuminating blue light penetrates the MP twice, the double MPOD (2MPOD) is the logarithm of the shading function divided by the reflectance in the original fundus image, according to the following equation, for each pixel of the image:

2MPOD=klogshadingfunction/measuredreflectance. (1)

The constant *k* is the ratio of the extinction of MP in the maximum at 460 nm and the extinction of MP at the wavelength of the blue light used^20^. In preliminary studies, values of MPOD were found comparable to the results obtained with other methods, if the ratio in Eq 1 is assumed to be a single optical density (OD). For this reason, all further calculations in our study have been considered as single OD. The advantage of this method is that the calculation of the MPOD by the ratio of the shading function and detected foveal reflectance acts as an internal normalization.[20]

### Spectral domain optical coherence tomography scan protocol and analysis

We used the Rtvue-100 system (Optovue Inc. Fremont, CA, USA) to analyze images with a signal strength indicator score greater than 45. An MM5 grid scan was performed to analyze macular volume, and a 3D reference grid scan was used to obtain en-face imaging. The Rtvue-100 device automatically calculated the retinal thickness precisely at the fovea (within 1 mm of the foveal center), parafovea (within a 1–3 mm ring from the fovea), and perifovea (within a 3–5 mm ring from the fovea). The en-face slice was performed at the ILM line level, and was 9–30 μm thick, as needed.

### Microperimetry protocol and analysis

Microperimetry was completed using the Nidek-MP1 (NAVIS software version 1.7.2, Nidek Technologies, Albignasego, Italy).[21–24] The following setting were used: red ring at 2° fixation target; monochromatic white background at 4 asb, Goldman III stimulus size with 200 ms projection time; 61 stimuli covering the central 10° with a radial grid pattern centered on the fovea; and 4-to-2 threshold strategy. During the test, the MP1 was provided with an automated tracking system to compensate for eye movements. The color image was acquired after the examination and was overlaid onto the retinal sensitivity points. Twenty minutes before the examination, one drop of 1% tropicamide and 2.5% phenylephrine was instilled into the eye to cause pupil dilatation. During the test, the contralateral eye was patched.

### Surgery

Small-gauge 3-port pars plana vitrectomy (PPV) was performed as follows: core vitrectomy, detachment of the posterior hyaloid, ERM-ILM peeling, control of the retinal periphery with external scleral indentation, and fluid–air exchange, air was the only type of tamponade used. The cataract surgery and intraocular lens implantation were performed at the same time in 8 of 15 iERM eyes and 12 of 15 FTMH eyes. In all other cases, the lens was transparent. One experienced staff member performed the operations.

### Statistical analysis

Statistical analysis was performed using Stata11 software (StataCorp. 2009, Stata Statistical Software: Release 11, College Station, TX). The unpaired *t* test was used to evaluate differences between the study groups. Pearson correlation coefficients (*r*) and linear and nonlinear (second-order model) regression models with analysis of variance were used to examine the relationships between mean MPOD and best-corrected visual acuity (BCVA) and macular sensitivity. Significance was defined as *P* < .05.

## Results

### Anatomical findings

There was no difference in the mean age of patients between the three groups (Table 1) (S1 Xlsx).

**Table 1:** Demographic characteristics of the study population.

	Control (30 eyes)	iERM (15 eyes)	FTMH (15 eyes)	*P* value (control vs iERM)	*P* value (control vs FTMH)	*P* value (iERM vs FTMH)
Age	66.6 (±7.4)	68.8 (±8.8)	66.5 (±8.7)	.3	.96	.47
BCVA (logMAR)	0.05 (±0.1)	0.7 (±0.3)	1.3 (±0.5)	**<0.001**	**<0.001**	**.0004**
MT (μm)	261.8 (±19.6)	403.0 (±78.4)	319.8 (±47.7)	**< .0001**	**< .0001**	**.0015**
MPOD mean (d.u.)	0.18 (±0.02)	0.15 (±0.04)	0.1 (±0.02)	**.0016**	**< .0001**	**.0002**
MPOD max (d.u.)	0.47 (±0.06)	0.51 (±0.13)	0.4 (±0.08)	.16	**.0020**	**.0094**
MPOD area (pixel)	77 628.8 (±8282.6)	55 121.23 (±18 054.7)	69 597.1 (±16 739.2)	**< .0001**	**.03**	**.03**
MPOD volume (d.u. x pixel)	14 338.3 (±2822)	8598.4 (±3438.0)	10 825.6 (±3329.7)	**< .0001**	**.0006**	.08
Macular sensitivity (db)	19.7 (±0.2)	8.1 (±3.8)	8.8 (±2.9)	**< .0001**	**< .0001**	.3

iERM = idiopathic epiretinal membrane; FTMH = full thickness macular hole; BCVA = best-corrected visual acuity; MT = macular thickness; MPOD = macular pigment optical density; d.u. = density units; db = decibels. Numbers in brackets are standard deviations. *P* values in bold indicate statistical significance.

### Baseline findings

At baseline, we reported significant differences in mean MPOD between the control, iERM and FTMH groups, in MPOD volume between the control vs iERM (*P<* 0.0016) and control vs FTMH groups (*P*<0.0001), iERM vs FTMH (*P*<0.0002); and in MPOD area between the control and iERM group (*P*<0.0001). We also found a larger MPOD max in the control compared to the FTMH group (*P*<0.0020), as well as a larger MPOD area in the control compared to the iERM group (*P*<0.0001) (Table 1) (S1 Xlsx).

### Changes after vitrectomy

**Fig 1:**
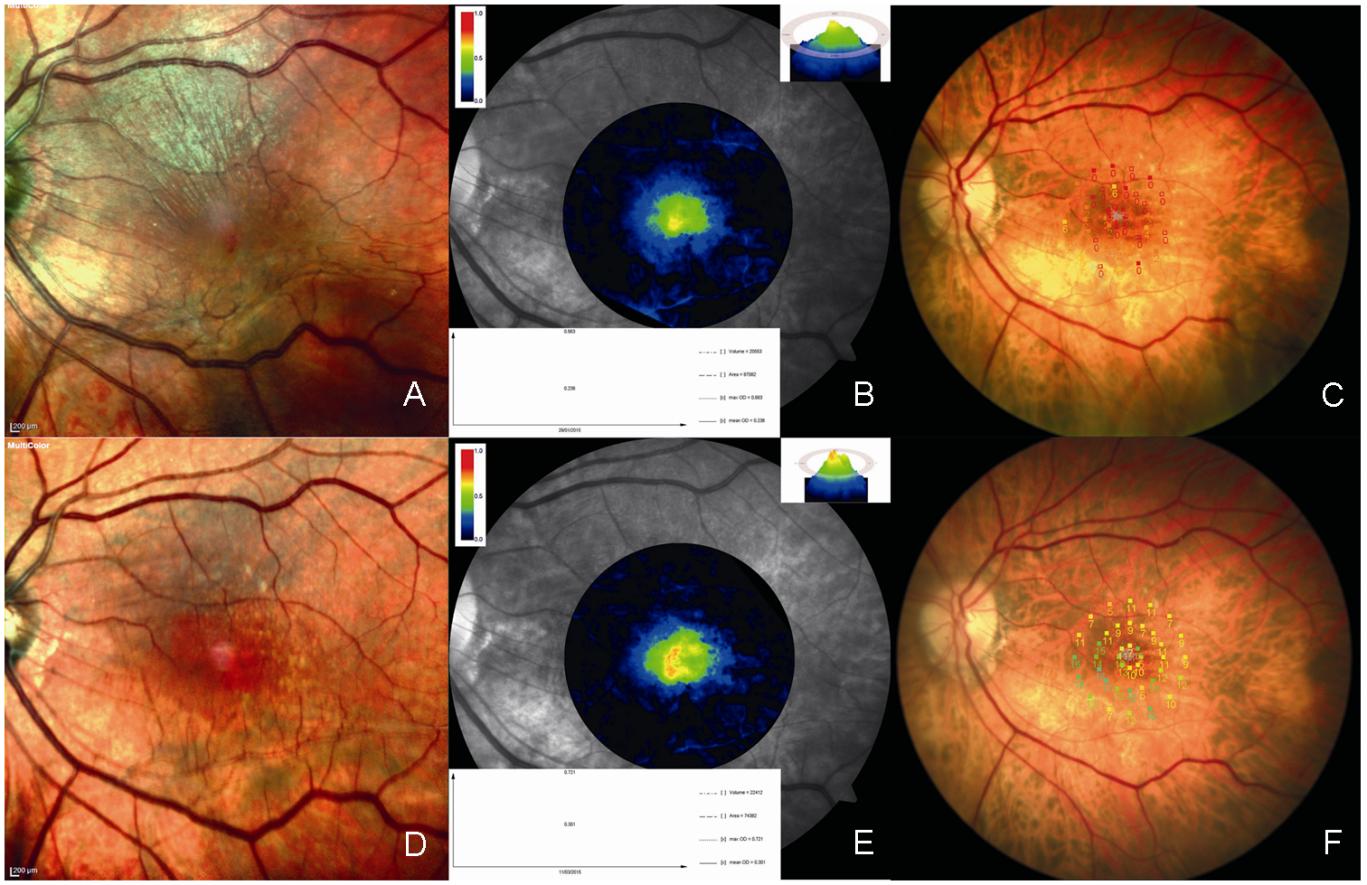
Macular pigment changes after ERM peeling. Multicolor imaging (A and D), MPOD level (B and E), and microperimetry (C and F) in the left eye of a 68-year-old woman with idiopathic epiretinal membrane at baseline (A–C) and at 6 months after vitrectomy (D–F). Multicolor imaging shows the disappearance of idiopathic epiretinal membrane following vitrectomy. The MPOD level increased after vitrectomy. Microperimetry shows an increase in macular sensitivity following vitrectomy.

**Fig 2:**
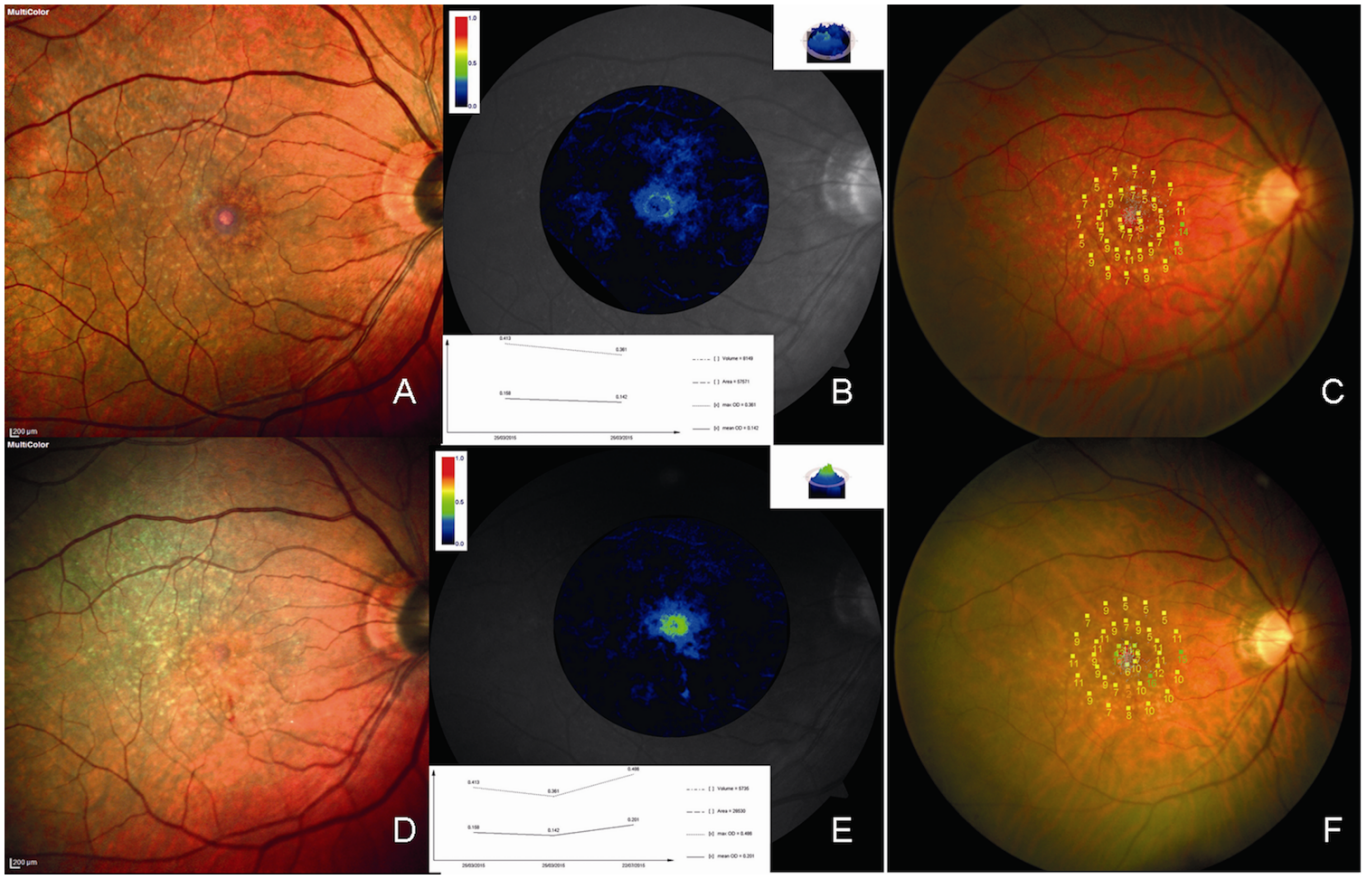
Macular pigment changes after ILM peeling in full-thickness macular hole. Multicolor imaging (A and D), MPOD level (B and E), and microperimetry (C and F) in the right eye of a 67-year-old woman with full-thickness macular hole at baseline (A–C) and at 6 months after vitrectomy (D–F). Multicolor imaging shows the full-thickness macular hole closed following vitrectomy. There is a small retinal hemorrhage inferior at the fovea. The MPOD level increased after vitrectomy. Microperimetry shows an increase in macular sensitivity following vitrectomy.

After vitrectomy and ERM-ILM peeling, the BCVA significantly increased in both iERM (*P*<0.0006) and FTMH group (*P*<0.0011) The most changes in MPOD were not statistically significant, other than MPOD volume after surgery in the FTMH group (*P*<0.01) (Table 2) (S1 Xlsx) (Figs 1 and 2). 13 of 15 patients (86.67%) with FTMH had complete closure of the hole, six months after surgery.

**Table 2:** Macular pigment optical density changes after ERM-ILM peeling.

	iERM		FTMH		*P* value (pre vs post)	
	*Preoperative*	*Postoperative*	*Preoperative*	*Postoperative*	*iERM*	*FTMH*
BCVA (logMAR)	0.7 (±0.3)	0.34 (±0.2)	1.3 (±0.5)	0.7 (±0.4)	**.0006**	**.0011**
MT (μm)	403.0 (±78.4)	354.6 (±66.6)	319.8 (±47.7)	262.0 (±9.3)	.07	**.0001**
MPOD mean (d.u.)	0.15 (±0.04)	0.2 (±0.1)	0.1 (±0.02)	0.1 (±0.01)	.08	>.9
MPOD max (d.u.)	0.51 (±0.13)	0.5 (±0.2)	0.4 (±0.08)	0.4 (±0.04)	.8	>.9
MPOD area (pixel)	55 121.23 (±18 054.7)	55 967.0 (±16 210)	69 597.1 (±16 739.2)	79 862.5 (±25 703.0)	.8	.2
MPOD volume (d.u. x pixel)	8598.4 (±3438.0)	10 066.0 (±3897.3)	10 825.6 (±3329.7)	14 569.1 (±4764.6)	.2	**.01**
Macular sensitivity (db)	8.1 (±3.8)	11.1 (±3.1)	8.8 (±2.9)	11.6 (±2.6)	**.025**	**.0095**

iERM = idiopathic epiretinal membrane; FTMH = full thickness macular hole; BCVA = best-corrected visual acuity; MT = macular thickness; MPOD = macular pigment optical density; d.u. = density units; db = decibels. Numbers in brackets are standard deviations. *P* values in bold indicate statistical significance.

### Correlations

We found a significant correlation between mean postoperative MPOD and BCVA in the iERM group (*r* = 0.739 *P* = 0.002), but not in the FTMH group *r* = 0.474 *P* = 0.342). Macular sensitivity was not significantly associated with mean MPOD before or after surgery (Table 3)(S1 Xlsx).

**Table 3:** Correlations between changes in MPOD and macular pigment variables after membrane peeling.

	MPOD mean before ERM-ILM	MPOD mean after ERM-ILM	MPOD mean before FTMH	MPOD mean after FTMH
Macular sensitivity (db) before ERM-ILM	*r* = 0.091 *P* = 0.746	*r* = 0.093 *P* = 0.742	–	–
Macular sensitivity (db) after ERM-ILM	*r* = 0.024 *P* = 0.964	*r* = –0.211 *P* = 0.451	–	–
BCVA (logMAR) before ERM-ILM	*r* = –0.391 *P* = 0.15	*r* = –0.001 *P* = 0.998	–	–
BCVA (logMAR) after ERM-ILM	*r* = –0.1 *P* = 0.85	*r* = 0.739 *P* = 0.002	–	–
Macular sensitivity (db) before FTMH	–	–	*r* = 0.274 *P* = 0.600	*r* = –0.47 *P* = 0.929
Macular sensitivity (db) after FTMH	–	–	*r* = 0.390 *P* = 0.445	*r* = 0.121 *P* = 0.820
BCVA (logMAR) before FTMH	–	–	*r* = 0.646 *P* = 0.166	*r* = 0.190 *P* = 0.718
BCVA (logMAR) after FTMH	–	–	*r* = 0.474 *P* = 0.342	*r* = –0.102 *P* = 0.848

MPOD = macular pigment optical density; ERM-ILM = idiopathic epiretinal membrane–internal limiting membrane peeling; FTMH = full thickness macular hole; BCVA = best-corrected visual acuity; MT = macular thickness; db = decibels.

## Discussion

Our results show that MPOD was significantly reduced in eyes with vitreoretinal interface syndromes compared with normal eyes. Several hypotheses can explain this result, as discussed below.

### MPOD in healthy eyes vs iERM or FTMH

The spatial distribution of MP is thought to due, at least in part, to the distribution of the cone photoreceptors, which decreases rapidly from the center of the fovea outward, or due to the foveal architecture, particularly the foveal width.[25,26] Using confocal and nonconfocal autofluorescence imaging and reflectance imaging, two recent studies in normal subjects found that about half of the subjects had a ring-like distribution superimposed on the flanks of a central peak.[27,28] One study also presented evidence that differences in MP distribution may be related to anatomical differences in the shape of the foveal depression.[28] This variability in the distribution of the MP has also been observed in monkeys by Snodderly et al.[29]

We found statistically significant differences in MPOD between healthy eyes and eyes with vitreoretinal interface syndromes (iERM or FTMH), but the etiology is probably different in these two conditions. In iERM, the MP can be obscured by the ERM in a shielding effect, but most often it is distorted by the constriction of the fovea induced by the iERM. In FTMH, there is a lack of MP in an area corresponding to the surface of the hole, due to the opening of the fovea and a centrifugal displacement of the MP.

### MPOD after macular peeling

We found an increase in mean MPOD, MPOD area and MPOD volume after ERM-ILM peeling in the iERM group and the FTMH group; however, only the change in MPOD volume after surgery in the FTMH group was statistically significant. For the iERM group, we hypothesize that the pre-surgical MPOD area constriction is similar to the constriction and thickening of the fovea induced by the contractile properties of iERM. After ERM peeling, there is an expansion of the macula and MP, and consequently of MP area and volume.

For the FTMH group, we hypothesize that closure of the surface of the hole leads to migration of cone photoreceptors and their axons, and consequently to recovery of MP in the foveal center and an increase of MPOD area and MPOD volume, as suggested by Shiragami et al.[30]. Jordan et al. report a significant increase in maximum MPOD and MPOD volume after successful PPV in patients with macular holes of stage 3 and 4, similar to our study.[31] Bottoni et al found an early increase in MPOD values during the first postoperative month after FTMH treated with 25-gauge PPV+ILM peeling, and that MPOD values did not change significantly throughout the follow-up period, similar to our results. They hypothesized that an early revisualization of MPOD after surgery occurred after the normal, early reapposition of the edges of the hole, where xanthophyll had been retracted before surgery.[32]

After successful macular hole closure, the structure of the retinal layer in the macula may not recover completely. Hangai et al reported that centrifugal enlargement of tiny interruptions in the external limiting membrane (ELM) occurs in macular hole closure if Muller cells’ cones are dragged away.[33] Because the ELM is formed in part by Muller cells, early ELM recovery might indicate early recovery of the retinal morphological structure, including the inner retinal layers. The ELM lines recovered earlier than the ellipsoid zone in the eyes of all patients: the ellipsoid zone recovery at 3 months postoperatively correlated significantly with ELM recovery at 1 month. The presence of a normal ellipsoid zone may indicate morphological and functional recovery of the photoreceptors as the MP in inner retinal layers recovers, and the recovery of the ellipsoid zone may determine most of the final functional improvement after surgery, as previously reported in literature.[32–35]

The inner retinal layers move to their normal position with time, and hyperautofluorescence can be seen through the damaged macula with decreasing pigment concentration after surgery. Shiragami et al reported that 46.2% of eyes showed hyperautofluorescence in the macular area 1 month postoperatively. This indicates that hyperautofluorescence in those eyes was caused by both the decreased MP and the RPE, which had normal metabolic function.[30]

Although we found increased macular sensitivity after surgery in both the iERM and FTMH groups, this increase was not statistically significant. Nor was there statistical significance associated with mean MPOD changes. These results support the idea that changes in MPOD do not show a direct correlation with the final functional outcome after surgery in patients with FTMH, as previously reported.[32] The reason for this could be that the mechanical microscopic trauma of ERM-ILM peeling caused by the use of intraocular forceps affects the outer retinal structure through the Muller cells.[36,37] Nevertheless, these changes seem to have no significant impact on postoperative functional outcome.[36]

### Correlation between MPOD and BCVA

Interestingly, we found a significant correlation between the mean postoperative MPOD and postoperative BCVA. We hypothesize that the postoperative increase in mean MPOD could be due to the unfolding and expansion of the fovea, and that postoperative increase in BCVA could be due to the restoration of the integrity of the external photoreceptor layers at the fovea, especially the inner segment/outer segment line, as suggested by Rii et al.[38] If displacement of the MP-containing inner retinal layers exists at the fovea soon after the operation, and if the RPE function is normal in the macular area, hyperautofluorescence may be concealed by MP.[30]

This hyperautofluorescence in the macular region is significantly associated with good visual prognosis. By contrast, late ELM recovery indicates insufficient recovery of the retinal structure, which may lead to late ellipsoid zone recovery, photoreceptor dysfunction at the fovea after macular hole surgery, and poor visual prognosis.[33] Therefore, an increased MPOD area, which is suggestive of a change in distribution, together with increased MPOD volume and mean MPOD after ERM-ILM peeling may be considered as a prognostic factor associated with a good visual prognosis, especially in patients with iERM.

### Limitations

One limitation of our study is the small number of patients with vitreoretinal interface syndrome. Another relates to the reflectometry technique itself, in which the absorption, reflection, and scattering are assumed to be homogenous across the area of the retina studied, which is a simplification.[39] Any reflection or scattering of light from the anterior part of the eye can influence MPOD readings, and there is need for target fixation by patients, which is not practical in patients with fixation loss.

### Conclusion

An increase in mean MPOD after ERM-ILM peeling seems to be associated with a good visual prognosis, especially in eyes with iERM. However, further studies are needed to investigate whether mean MPOD, MPOD volume, and MPOD area are prognostic factors after PPV performed for vitreoretinal interface syndrome, and to assess their associations with general retinal sensitivity.

## Supporting information

S1 Xlsx Data of study patients. Demographic and preoperative and postoperative macular pigment optical density of patients affected by idiopathic epiretinal membrane or full-thickness macular hole.
(XLS)
